# Supplementary material for: Mechanisms of Ca2+/calmodulin-dependent kinase II activation in single dendritic spines
Source: Nat Commun. 2019 Jun 25;10:2784. doi: 10.1038/s41467-019-10694-z (PMC6592955; doi:10.1038/s41467-019-10694-z)
Supplement: Supplementary file 1 — Supplementary Information [file 41467_2019_10694_MOESM1_ESM.docx]

**Supplementary Information**

**Mechanisms of Ca^2+^/Calmodulin-dependent kinase II activation in single dendritic spines**

Jui-Yun Chang^1,2^, Yoshihisa Nakahata^2^, Yuki Hayano^2^ and Ryohei Yasuda^2^

^1^Department of Biochemistry, Duke University, Durham, NC 27707, USA

^2^Neuronal Signal Transduction Group, Max Planck Florida Institute for Neuroscience, Jupiter, FL 33458, USA

**Supplementary Figure 1: Effects of the overexpression level of CaM-CaMKIIα association sensor on the association of mEGFP-CaMKIIα and mCherry-CaM**

Fluorescence intensity of mEGFP-CaMKIIα (**a**) or mCherry-CaM (**b**) at proximal apical dendrites were used to quantify the overexpression level.

(**a**) Relative expression level of mEGFP-CaMKIIα^WT^ and mEGFP-CaMKIIα^T286A^ (ref 6). There were weak to no correlations between changes in CaMKIIα-CaM association and expression level of mEGFP-CaMKIIα (r = -0.53 for CaMKIIα^WT^ (p < 0.05) and r = -0.09 for CaMKIIα^T286A^ (p > 0.05)).

(**b**) Relative expression level of mCherry-CaM. There was no significant correlation between changes in CaMKIIα-CaM association expression level of mCherry-CaM (r = 0.35 for CaMKIIα^WT^ (p > 0.05) and r = -0.2 for CaMKIIα^T286A^ (p > 0.05)).

**Supplementary Figure 2: CaMKIIα-CaM association at a near physiological temperature (34-35 °C)**

Averaged changes in CaMKIIα^WT^-CaM (A; n =16 spines/5 neurons) and CaMKIIα^T286A^-CaM association (B; n = 19 spines/4 neurons) in the stimulated spine (black) and adjacent dendrite (blue) during sLTP induction (0.49 Hz, 30 pulses). The orange curves are obtained by curve fitting of a function: *B*(*t*) = *B*_0_ exp(– *t* / τ) + *c.* The decay time constants are obtained as τ = 0.4 ± 0.5 s for CaMKIIα^WT^ (**a**) and τ = 0.3 ± 0.1 s for CaMKIIα^T286A^ (**b**). The left panels are expanded views of the right panels. All data are shown in mean ± sem, and sem of time constants is obtained by bootstrapping.
